# Supplementary material for: Discrimination of Isointense Bitter Stimuli in a Beer Model System
Source: Nutrients. 2020 May 27;12(6):1560. doi: 10.3390/nu12061560 (PMC7352581; doi:10.3390/nu12061560)

Supplementary information for Higgins and Hayes 2020

Supplementary Table 1: Mean ratings and standard deviation (SD) of remembered liking/disliking ratings for food and beverage items in the practice LAM ratings. Note: The n for each item varies because participants were allowed to skip and item if they were not familiar with a product.

Item	n	mean	SD (±)
Cotton Candy	81	59.1	16.0
Cola (e.g., Pepsi, Coke)	81	68.3	17.0
Black coffee	78	55.9	24.1
Coffee with creamer	79	65.9	22.9
Hot dogs	79	65.3	17.0
Broccoli	77	72.6	12.5
Skim milk	79	58.0	18.1
Milk chocolate	80	74.3	15.6
Cottage cheese	76	56.2	21.6
Oysters	74	49.4	25.4
Mozzarella cheese	81	77.1	12.6

Photograph of Beer Samples as Presented under Red Lighting

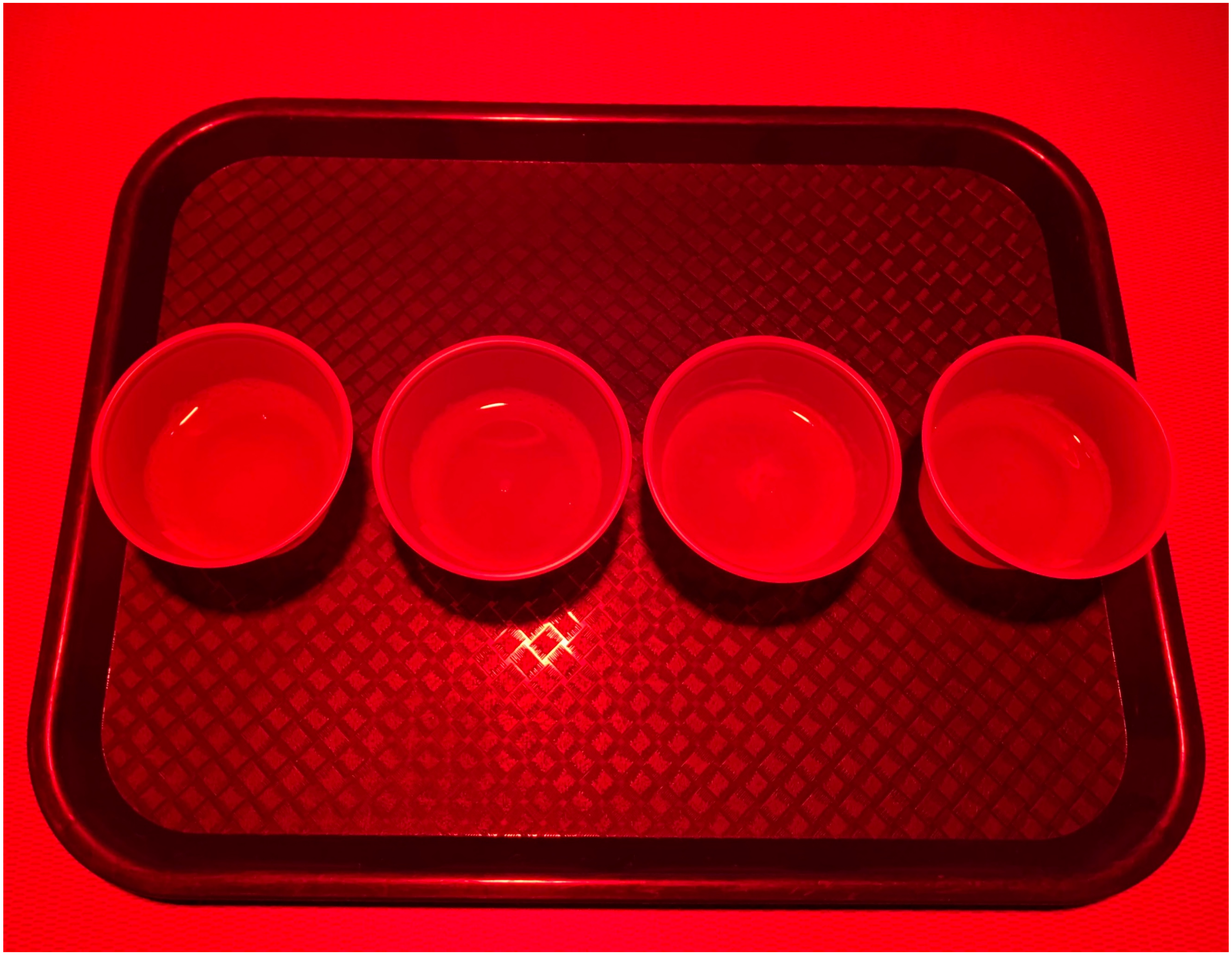

Supplement: Supplementary file 1 [file nutrients-12-01560-s001.pdf]
